# Association between Magnesium Depletion Score and Advanced Cardiovascular-Kidney-Metabolic Syndrome Stages in US Adults

**DOI:** 10.7150/ijms.115217

**Published:** 2025-06-23

**Authors:** Juan Tian, Xiaoyu Ding, Xiaoying Ren, Guang Wang, Wei Wang, Jia Liu

**Affiliations:** 1 Department of Endocrinology, Beijing Chao-Yang Hospital, Capital Medical University, No. 8, Gongti South Road, Chaoyang District, Beijing 100020, China.; 2 Department of Urology, Beijing Chao-Yang Hospital, Capital Medical University, No. 8, Gongti South Road, Chaoyang District, Beijing 100020, China.

**Keywords:** cardiovascular-kidney-metabolic syndrome, magnesium, magnesium depletion score, cross-sectional study

## Abstract

**Background:** Cardiovascular-kidney-metabolic (CKM) syndrome is highly prevalent. Advanced CKM stages have the potential for multiorgan disease, premature mortality, and excess morbidity. Magnesium deficiency has a prominent impact on the components of CKM syndrome. However, the relationship between magnesium depletion score (MgDS) and the risk of developing advanced stages of CKM syndrome is still unclear.

**Methods:** We used data from the National Health and Nutrition Examination Survey (NHANES) from 1999 to 2018. To investigate the relationship between MgDS categories and advanced CKM stages, we employed weighted multivariable logistic regression. Stratified and interaction analysis was conducted to find whether some demographics and lifestyle factors modified the association. Restricted cubic spline (RCS) analysis was implemented to investigate the dose-response relationships. Additionally, receiver operating characteristic (ROC) curve was plotted to assess the diagnostic accuracy of MgDS for advanced CKM stages.

**Results**: The study population comprised 18,038 participants aged 20 to 79 years. The multivariable logistic regression suggested that high MgDS levels (MgDS = 2 and ≥ 3) were associated with higher odds of having advanced CKM stages compared with low MgDS level (MgDS = 0) in all adjusted models (*P* < 0.05). Stratified and interaction analysis indicated that PIR had significant effects on the association (*P* for interaction < 0.05). The RCS regression analyses demonstrated a positive linear association between MgDS and the incidence of advanced CKM stages (P for overall = 0.0003, P for nonlinear = 0.1374). The MgDS predicted the area of the ROC curve of 0.7577 (*P* < 0.05) and the optimal cutoff value was 2.

**Conclusions:** Magnesium-depleted state as quantified by MgDS (MgDS ≥ 2) is a significant risk factor for advanced CKM stages, which suggests that identifying magnesium deficiency and improving the nutritional status of magnesium might mitigate the risk of advanced CKM stages.

## Introduction

Cardiovascular-kidney-metabolic (CKM) syndrome was defined as a systemic disorder by the American Heart Association (AHA), which is attributable to the pathophysiological connections among metabolic risk factors, chronic kidney disease (CKD), and cardiovascular disease (CVD) 1. To delineate the progressive nature of CKM syndrome, it was further categorized into stages: stage 0, absence of CKM risk factors; stage 1, excess and/or dysfunctional adiposity; stage 2, presence of metabolic risk factors, moderate- to high-risk CKD, or both; stage 3, presence of subclinical CVD; and stage 4, presence of clinical CVD 1. Recent research indicated that there is a high incidence of CKM syndrome in adults, with a higher burden reported among those with insulin resistance, unfavorable social risk profile or social determinants of health 2-4. Poor CKM health has been demonstrated to have multisystem consequences that extend beyond the simple sum of its components. These consequences may include multiorgan disease, premature mortality, and excess morbidity, which can result in high health care expenditures 1, 5. There is a critical need to screen for exposures most relevant to CKM health and to establish life-course interventions aimed at averting the progression of CKM syndrome and related outcomes.

Magnesium is essential for the physiological functions of nearly all organs in the human body. Beyond its foundational roles in RNA, DNA, and protein synthesis, magnesium plays a role in various biological processes, including the metabolism of lipids, glucose, bone and calcium (Ca), and the function of the immune system 6, 7. Magnesium deficiency is commonly seen in cardiovascular, renal, neurological, psychiatric, and metabolic diseases 8-12. Recent clinical and preclinical studies have suggested potential therapeutic effects of magnesium supplementation in various chronic conditions, including hypertension, stroke, heart failure, diabetes, and CKD 13.

Nonetheless, there is an absence of standardized methods for the precise evaluation of magnesium status. Although serum magnesium is the most commonly used clinical test, it does not always accurately reflect the body's overall magnesium levels, given that less than 1% of magnesium is present in serum 14. While 24-hour urinary magnesium concentrations can help evaluate magnesium status, they may be unreliable in patients with impaired kidney function and can be affected by diet and certain medications like proton pump inhibitors (PPIs) and diuretics 6. The magnesium tolerance test (MTT) is a more sensitive assessment 15. Due to time consuming and labor burden of conducting the MTT, this test is rarely used in clinic. The magnesium depletion score (MgDS) has been demonstrated to be a more accurate and effective predictor of magnesium deficiency status, which takes into account four key factors that influence the absorption and excretion of dietary magnesium, including diuretics and PPI use, deterioration in kidney function, and alcohol consumption 16. Individuals with a higher MgDS indicate a more severe nutritional state of magnesium shortage.

To our knowledge, there is no study focused on the association between MgDS and advanced CKM stages. It is necessary to identify the contribution of MgDS to advanced stages of CKM syndrome, which will help promote screening for magnesium deficiency and inform future development of strategies to prevent CKM progression. We conducted a cross-sectional study using data from the National Health and Nutrition Examination Survey (NHANES) 1999-2018 to explore the relationship between MgDS and advanced CKM stages.

## Materials and Methods

### Study population

The data for this cross-sectional study were obtained from the NHANES website (https://www.cdc.gov/nchs/nhanes/index.htm). NHANES is a large-scale nationwide survey with a complex, stratified, multistage probability sampling methodology, which is designed to assess the health and nutritional conditions of the general US population 17. The research was approved by the Institutional Review Board of the National Center for Health Statistics (NCHS), and written informed consent was obtained from all participants or their guardian prior to data collection.

**Figure 1:**
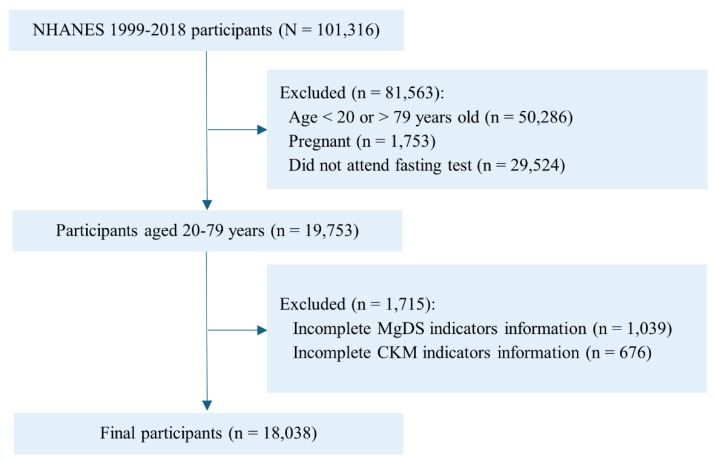
Flowchart of participant selection from NHANES 1999 to 2018. Abbreviations: NHANES, National Health and Nutrition Examination Survey; CKM, cardiovascular-kidney-metabolic; MgDS, magnesium depletion score.

In this study, we selected 101316 participants from 10 cycles of NHANES data (1999-2018). A total of 19753 individuals aged 20 to 79 years were selected after excluding participants who were pregnant and did not attend fasting test in the mobile examination center. We further excluded 1715 participants who did not have sufficient information to determine MgDS (n=1039) and CKM syndrome (n=676). Ultimately, 18038 adults were enrolled in the final analysis (**Figure 1**).

### Magnesium depletion score calculation

The MgDS was calculated by summing points from the following 4 scores: (1) current use of diuretics was scored 1 point; (2) current use of proton pump inhibitor (PPI) was scored 1 point; (3) an estimated glomerular filtration rate (eGFR) more than 90 mL/min/1.73 m^2^ was scored 0 point, an eGFR between 60 mL/min/1.73 m^2^ and 90 mL/min/1.73 m^2^ was scored 1 point, while an eGFR less than 60 mL/min/1.73 m^2^ was scored 2 points; and (4) heavy drinking (> 1 drink/day for women and > 2 drinks/day for men, according to 2015-2020 Dietary guidelines for Americans) was scored 1 point 16. The eGFR was calculated using the 2021 race- and ethnicity-free Chronic Kidney Disease Epidemiology Collaboration creatinine equation 18. According to previous validation studies 19, 20 and the limited sample size in higher MgDS categories (scores 3-5) across CKM stages within our study, MgDS was categorized into 4 groups: MgDS = 0, MgDS = 1, MgDS = 2, and MgDS ≥ 3.

### CKM syndrome stages

The definition of CKM syndrome stages is according to the criteria from Li J *et al.* 3. Briefly, the stages are delineated as follows:

Stage 0, individuals with normal body mass index (BMI < 25 kg/m^2^, or < 23 kg/m^2^ if Asian ancestry), normal waist circumference (waist circumference < 88/102 cm in female/male or if Asian ancestry < 80/90 cm in female/male), without other metabolic disorders (prediabetes, diabetes, hypertension, hypertriglyceridemia [≥ 135 mg/dL], metabolic syndrome), low-risk CKD, predicted 10-year CVD risk < 20%, and no evidence of clinical CVD (history of chronic heart failure, coronary heart disease, heart attack, or stroke).

Stage 1, individuals with overweight/obesity (BMI ≥ 25 kg/m^2^, or ≥ 23 kg/m^2^ if Asian ancestry), abdominal obesity (waist circumference ≥ 88/102 cm in female/male or if Asian ancestry ≥ 80/90 cm in female/male), or prediabetes (fasting blood glucose of 100-124 mg/dL or glycosylated hemoglobin A1c of 5.7%-6.4% and without self-reported diagnosis of diabetes, use of insulin, or oral hypoglycemic agents), and meeting all of the following conditions: without other metabolic disorders, low-risk CKD, predicted 10-year CVD risk < 20%, and no evidence of clinical CVD.

Stage 2, individuals with any of the four metabolic disorders (hypertriglyceridemia, hypertension, diabetes, metabolic syndrome), or moderate-to-high-risk CKD, and no evidence of subclinical CVD (very high-risk CKD, predicted 10-year CVD risk ≥ 20%) or clinical CVD.

Stage 3, subclinical CVD with any of the seven metabolic disorders (overweight/obesity, abdominal obesity, prediabetes, diabetes, hypertension, hypertriglyceridemia, metabolic syndrome) or moderate-to-high-risk CKD, and no evidence of clinical CVD.

Stage 4, clinical CVD with any of the seven metabolic disorders (overweight/obesity, abdominal obesity, prediabetes, diabetes, hypertension, hypertriglyceridemia, metabolic syndrome) or CKD.

Participants were also classified into 2 groups: nonadvanced (CKM stages 0, 1, or 2), advanced (CKM stages 3 or 4). Metabolic syndrome (MetS) was diagnosed according to the NCEP-ATP III report 21. CKD was defined as moderate- to high-risk or high-risk levels of CKD according to Kidney Disease Improving Global Outcomes (KDIGO) 2021 22. We calculated the 10-year CVD risk through the AHA PREVENT equations 23.

Recent studies using the NHANES database have applied CKM definitions according to the AHA Presidential Advisory Statement on CKM Syndrome 1. For example, Zhu R *et al.* (n=29722) used 10 NHANES cycles with modified criteria from Aggarwal *et al.*, which is similar to our definition but differs in age cut-offs for 10-year CVD risk estimation 4, 24. Notably, Tu D *et al.* (n=12245) employed Framingham risk score for 10-year CVD risk prediction 25, 26. Both Chen Y *et al.* (n=21609) and Wu S *et al.* (n=1889) adopted definitions aligned with that used in our study 27, 28. Collectively, while there are slight variations in the evaluation of CKM stages across these studies, our research maintains methodological consistency with established definitions, providing a robust framework for understanding CKM syndrome.

### Covariate assessment

Demographic covariates in this study included age, sex (male, female), race and ethnicity (Mexican American, non-Hispanic Black, non-Hispanic White, and other [other Hispanic or other race, including multiracial]), education level (> High school, High school, < High school), and poverty income ratio (PIR) (< 1.3, 1.3-3.5, > 3.5). Examination data included BMI, waist circumference. Laboratory data included triglycerides (TG), total cholesterol (TC), high-density lipoprotein cholesterol (HDL-c), low-density lipoprotein cholesterol (LDL-c), eGFR, HOMA-IR [HOMA-IR = Fasting Glucose (mmol/L) × Fasting Insulin (uU/mL) / 22.5], glycated hemoglobin A_1c_ (HbA_1c_), serum calcium (Ca). Dietary data included magnesium intake. Lifestyle data included current smoker (yes, no), current drinker (yes, no) and physical activity (active, refers to at least 150 minutes per week of moderate-intensity activity or 75 minutes per week of vigorous-intensity activity; inactive indicates less than these levels).

### Statistical analysis

The data were processed according to NHANES analytical guidelines. All analyses used appropriate sample weights and strata because of the complex, multistage, sampling design of NHANES 29. For continuous variables, statistics were described using the weighted mean ± standard error (SE), and differences between groups were investigated using analysis of variance (ANOVA) or Student's t-test. Categorical variables were described using frequency with weighted percentages, and intergroup differences were assessed using the chi-square test. The relationship between MgDS and advanced CKM Stages was analyzed using weighted multivariable logistic regression. Model 1 was adjusted for age, sex, race, education and PIR. Model 2 was adjusted for the variables in model 1 plus BMI, TG, HDL-c, LDL-c, HOMA-IR, HbA_1c_, serum calcium (Ca), magnesium intake. Model 3 was adjusted for the variables in model 2 plus current smoker, current drinker, physical activity. The missing data for the participants in this study were shown in **Table S1**. Two sensitivity analyses were performed to address missing data. First, multiple imputation was conducted on the data using the “jomo” package 30. In addition, we excluded individuals with any missing values to verify the robustness of the results. The test for multicollinearity (**Table S2**) showed that the variance inflation factor (VIF) for each covariate was less than 5, indicating the absence of substantial multicollinearity among the covariates 31.

Given that demographics and lifestyle factors were different in MgDS and advanced CKM Stages, stratified analyses were also performed. Restricted cubic spline (RCS) analysis was conducted to investigate the potential nonlinear relationship between MgDS and advanced CKM Stages. To determine the optimal number of knots, we compared models with 3-6 knots using both Akaike Information Criterion (AIC) and Bayesian Information Criterion (BIC). Given the large-scale nature of our dataset, we prioritize the lowest BIC value for knots selection to reduce the risk of overfitting and improve the interpretability of the model. The results of AIC and BIC calculations and the final selected number of knots are detailed in **Table S7**. Receiver operating characteristic (ROC) curve and the area under the curve (AUC) were used to evaluate the predictive efficacy of MgDS on patients with advanced CKM Stages.

All statistical analyses were performed using R software (version 4.4.2). A 2-sided *P* < 0.05 was considered significant.

## Results

### Participant characteristics

A total of 18038 participants were included in the study. Baseline characteristics, as presented in **Table 1** according to CKM stages, revealed that participants with advanced CKM stages were more likely to be older, male, of non-Hispanic black and white ethnicity, and with lower education and family income levels. Compared with individuals with nonadvanced CKM stages, participants with advanced CKM stages exhibited higher BMI, higher waist circumference, higher TGs, lower HDL-c, lower eGFR, higher HOMA-IR, higher HbA_1c_, higher serum Ca levels, and consume less magnesium. However, a notable observation was the significantly lower levels of TC and LDL-C in participants with advanced CKM stages. This finding might be attributable to the use of lipid-lowering medications. The advanced CKM group had more current smokers and non-current drinkers, more diuretic and PPI users, fewer heavy drinkers, and higher eGFR scores. Specifically, the advanced CKM group showed unfavorable MgDS levels. In contrast to the nonadvanced CKM group, participants with MgDS categories of 0 or 1 were less prevalent in the advanced CKM group, whereas those with MgDS categories of 2 or ≥ 3 were more prevalent in the advanced CKM group (all *P* < 0.05). The baseline characteristics of individuals after multiple imputation and excluding those with any missing values are also detailed in **Table S3** and **Table S5**.

### Prevalence of CKM conditions in each MgDS category

**Figure 2:**
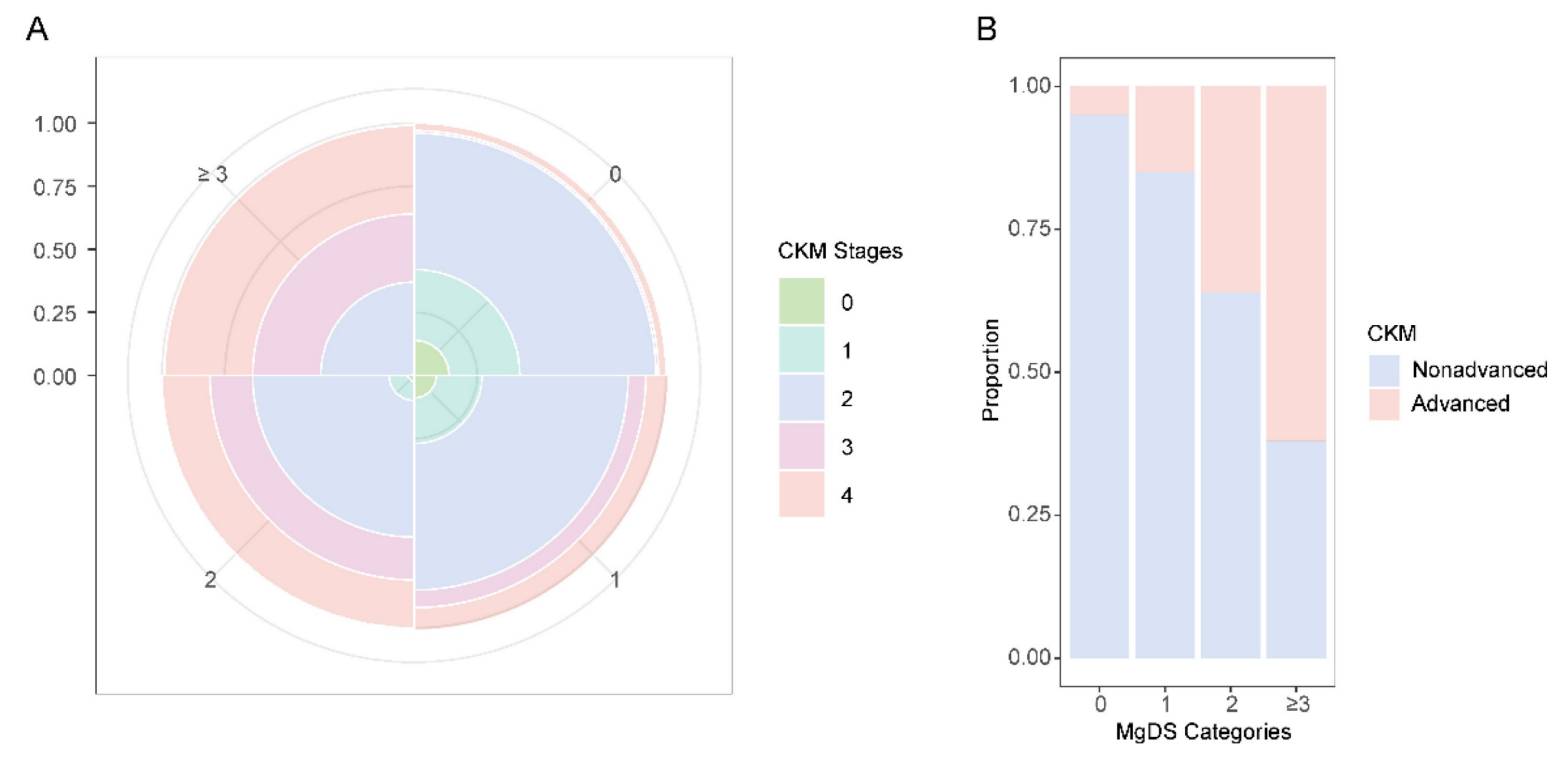
Proportional Rose plot and Bar chart of CKM conditions by MgDS categories. (A) Rose plot showing the prevalence of CKM stages (0-4) in each MgDS category. (B) Bar chart comparing the prevalence of CKM (nonadvanced and advanced) in each MgDS category.

In overall participants, the prevalence of CKM stage 2 accounted for the largest proportion in MgDS levels 0, 1, and 2. Advanced CKM stages (stage 3 or 4) were the most prevalent CKM condition in high MgDS level (≥ 3). The prevalence of advanced CKM stages showed an increasing trend with rising MgDS levels (**Figure 2**).

### Multivariable logistic regression analysis

The survey-weighted multivariable regression analyses showed a positive association between MgDS categories and the odds of advanced CKM stages. Compared with low MgDS level (MgDS = 0), the odds ratios (OR) for advanced CKM stages among participants with high MgDS levels (MgDS = 2 and ≥ 3) were 2.31 (95%CI 1.82-2.94), 4.45 (95%CI 3.32-5.98) in the minimally adjusted Model 1 (all *P* < 0.05). This association remained significant for the MgDS = 2 and MgDS ≥ 3 groups in the moderately adjusted Model 2 (OR 2.18, 95%CI 1.70-2.80; OR 3.68, 95%CI 2.68-5.06) and the fully adjusted Model 3 (OR 2.33, 95%CI 1.74-3.14; OR 3.41, 95%CI 2.28-5.11) (all *P* < 0.05) (**Table 2**). As demonstrated in **Table 2**, the odds of advanced CKM stages showed an increasing trend with elevated MgDS levels (*P* for trend < 0.05).

### Stratified and interaction analysis

**Figure 3:**
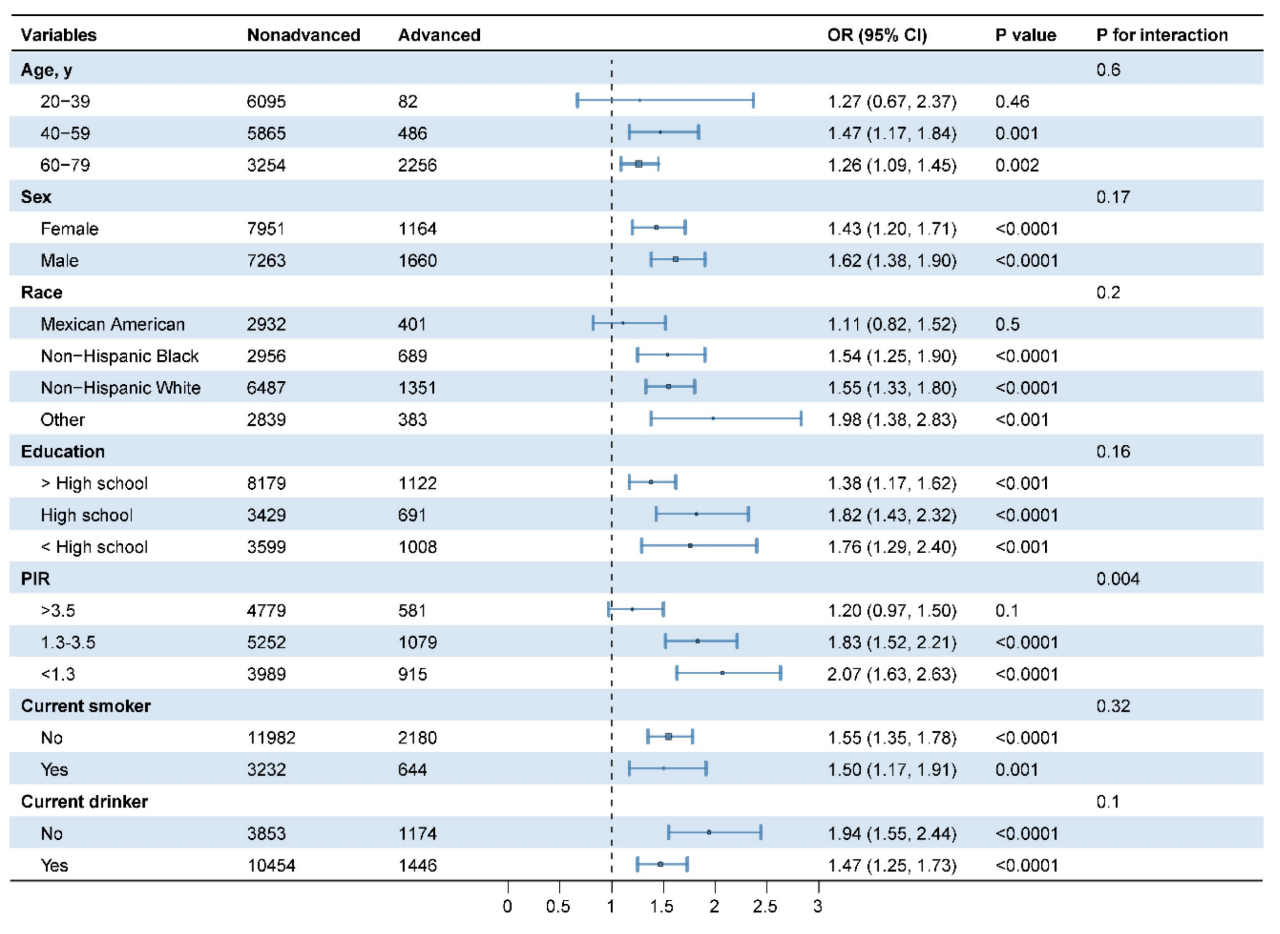
Subgroup analysis for the association between MgDS and advanced CKM stages. Adjusted for BMI, TGs, HDL-c, LDL-c, HOMA-IR, HbA_1c_, Ca, magnesium intake, physical activity.

Stratified and interaction analyses were conducted to assess the potential modification of demographic and lifestyle variables (age, sex, race, education, PIR, smoking, and drinking status) on the relationship between MgDS and the incidence of advanced CKM stages (**Figure 3**). After adjusting for potential confounders, PIR had significant effects on the association (*P* for interaction < 0.05). Among the three PIR groups, participants in lower PIR groups with elevated MgDS demonstrated significantly greater odds of advanced CKM stages. In the 1.3 ≤ PIR ≤ 3.5 group, the OR was 1.83 (95%CI 1.52-2.21, *P* < 0.05). The effect was even stronger in the lowest PIR group (PIR < 1.3), where the odds ratio was 2.07 (95%CI 1.63-2.63, *P* < 0.05). Conversely, for participants in the highest PIR group (PIR > 3.5), the association between MgDS and advanced CKM stages was not statistically significant (OR 1.20, 95%CI 0.97-1.50, *P* = 0.1). Other variables demonstrated no significant interactions (all *P* for interaction > 0.05) (**Figure 3**).

### Sensitivity analysis

We conducted two sensitivity analyses to validate the robustness of our results. First, multiple imputation techniques were utilized to address missing values, and multivariable logistic regression analysis was conducted to explore the relationship between MgDS and advanced CKM stages. In addition, we conducted an analysis excluding individuals with any missing values. The results consistent with the primary findings (**Table S4** and **Table S6**).

### RCS analysis

**Figure 4:**
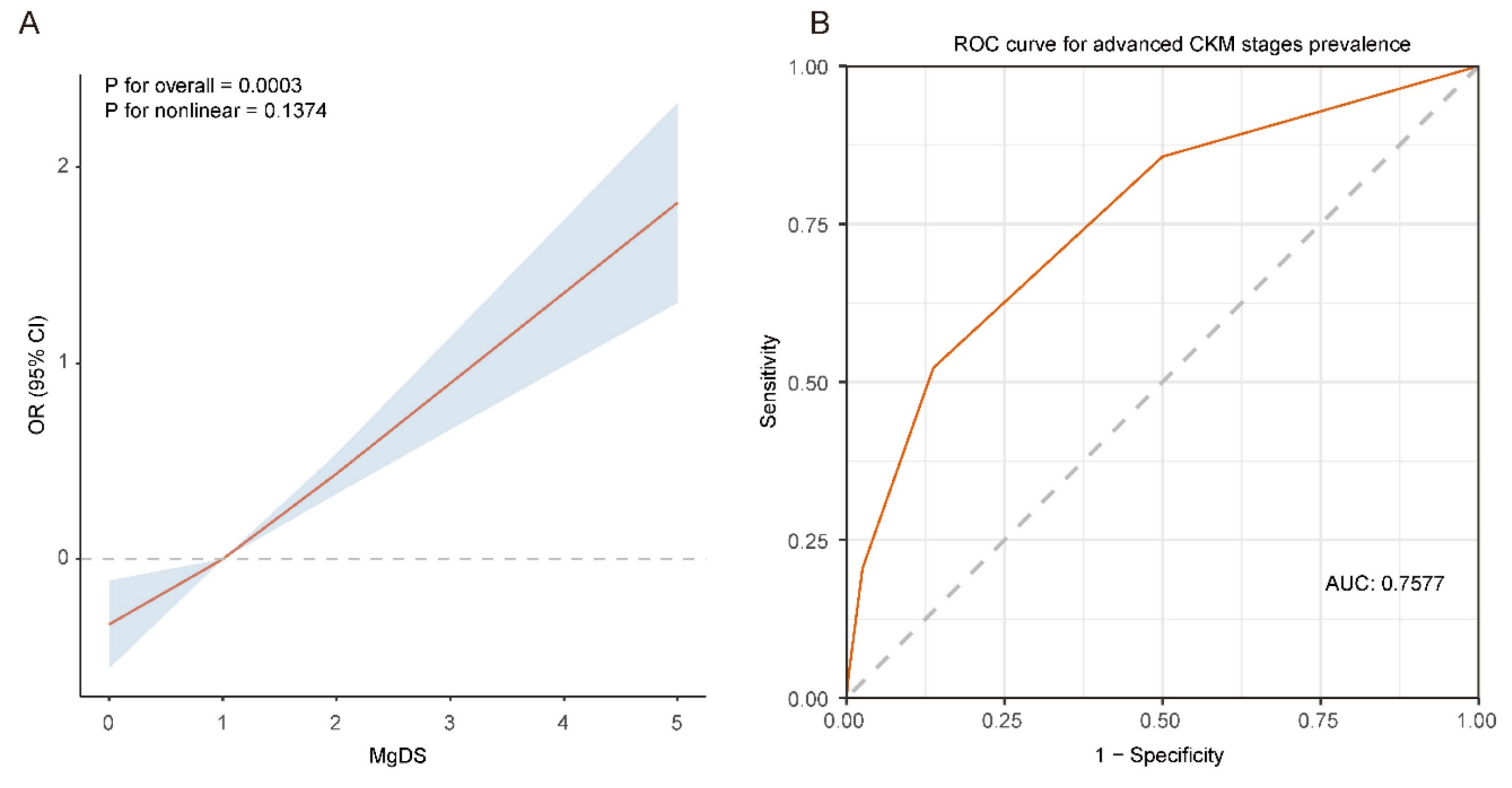
The RCS analysis and ROC curve between MgDS and risk of advanced CKM stages. (A) The RCS analysis between MgDS and risk of advanced CKM stages. The model was adjusted for age, sex, race, education, PIR, BMI, TGs, HDL-c, LDL-c, HOMA-IR, HbA_1c_, Ca, magnesium intake, current smoker, current drinker, physical activity. (B) The ROC curve of MgDS for predicting the risk of advanced CKM stages.

To further explore the relationship between MgDS and advanced CKM stages, we conducted RCS analysis with 3 knots. The results indicated that elevated MgDS showed a positive linear relationship with the OR of advanced CKM stages after full adjustment for confounders (P for overall = 0.0003, P for nonlinear = 0.1374) (**Figure 4A**).

### ROC analysis

ROC analysis was utilized to assess the predictive capability of MgDS on patients with advanced CKM stages (**Figure 4B**). The area under the ROC curve, as predicted by MgDS, was 0.7577 (95% CI 0.7515-0.7640, *P* < 0.05). The result revealed that MgDS had statistically significant diagnostic capability in the prediction of advanced CKM stages (AUC > 0.5). The optimal cutoff value for MgDS was 2, suggesting the potential predictive value for the detection of advanced CKM stages when the MgDS is greater than 2. The corresponding sensitivity and specificity were 52.2% and 86.3%, respectively.

## Discussion

Using a nationally representative sample of US adults, our study indicated that elevated MgDS levels (MgDS ≥ 2) were associated with an increased probability of advanced CKM stages (CKM stages 3 or 4). After fully adjusting for multiple variables, the positive correlation between MgDS and advanced CKM stages remained significant. Furthermore, we observed a linear relationship between MgDS and the odds of advanced CKM stages. In addition, ROC curve revealed that MgDS had diagnostic capability in distinguishing patients with advanced CKM stages from those with nonadvanced CKM stages when the MgDS is greater than 2. These findings highlight that magnesium depletion may be a key driver of advanced CKM morbidity and revealed the predictive role of MgDS in identifying high-risk individuals for CKM syndrome.

A growing body of research has documented that hypomagnesemia is prevalent among the general population, with evidence suggesting that individuals with lower magnesium levels are at a heightened risk for major components of CKM syndrome, including obesity, diabetes, hypertension, MetS, CVD, and CKD 8, 32-37. MgDS was recently proposed as a simpler and more precise alternative indicator for predicting magnesium deficiency. Research has indicated that an adverse MgDS acts as an independent risk component for MetS and diabetes 38, 39. A comprehensive cross-sectional and longitudinal study revealed that individuals with elevated MgDS levels exhibited an increased likelihood of CVD, accompanied by heightened mortality risk among patients with CVD 20. Another study from NHANES indicated that higher MgDS levels independently predict an elevated risk of CVD and longitudinal mortality among US adults with diabetic disease kidney (DKD) 40. Moreover, one study suggested that higher MgDS is strongly associated with an elevated risk of all-cause and cardiovascular mortality in participants with hypertension 41. Given these findings, investigating the association between MgDS and CKM syndrome is urgently needed. In this study, we revealed a positive association between MgDS and advanced CKM stages, which aligned with previous literature exploring the impact of MgDS on various components of CKM syndrome, including metabolic disorders and cardiovascular outcomes. Hence, our findings emphasize the importance of screening for magnesium status and imply that interventions aimed at improving the nutritional status of magnesium need to be carried out among magnesium-depleted populations (MgDS ≥ 2) in order to mitigate the risk of advanced CKM stages.

Subgroup analysis revealed that PIR had a notable impact on the relationship between MgDS and the occurrence of advanced CKM stages. Individuals in the lower PIR group with elevated MgDS showed more pronounced risks of advanced CKM stages. Research revealed that adults with low-income levels tend to have low intakes of magnesium-rich foods, which can contribute to magnesium-depleted nutritional status 42. Our finding imply that we ought to prioritize the issue of magnesium deficiency and its associated risk of advanced CKM stages in low-income populations.

The exact mechanism between magnesium deficiency and advanced CKM stages is not yet clear, there are several potential explanations. First, magnesium plays an important role in heart function through its substantial vasodilatory effect 43, 44. Magnesium deficiency promotes oxidative stress notably in endothelial cells, resulting in increased reactive oxygen species (ROS) and cytotoxicity 45. Low magnesium-induced oxidative stress induces the endothelium to develop a state of permanent inflammation, which is marked by increased NFκB activitiy 46. Under the condition of local inflammation, the vessel wall will recruit monocytes and trigger the proliferation and migration of vascular smooth muscle cells, resulting in atherosclerosis, thrombosis and CVD. Second, low serum magnesium levels induced chronic inflammation is associated with vascular calcification, which may result in CKD and CVD 6. Furthermore, insulin receptors (IR) are part of the family of tyrosine kinase receptors, and the kinase function is dependent on the binding of magnesium 47. In low magnesium conditions, diminished IR signal transduction may contribute to the development of diabetes mellitus by increasing insulin resistance. Consequently, magnesium deficiency may contribute to the advancement of CKM syndrome.

Utilizing a large sample population from NHANES, this study made a significant contribution by investigating the association between MgDS and advanced CKM stages for the first time. Our findings underscored significant disparities in the prevalence of advanced CKM stages across MgDS levels. These findings highlight the necessity for targeted screening and prevention strategies, including magnesium supplementation, among individuals at high risk of magnesium deficiency (i.e., with MgDS ≥ 2), to avert the development of advanced CKM stages. Of note, low family income populations deserve special attention and should be prioritized for intervention.

This study had several limitations. First, due to the cross-sectional nature of the design, it was not possible to establish a causal relationship between MgDS and advanced CKM stages. Second, CVD-related data was self-reported by participants in NHANES, which may slightly deviate from the actual incidence rates. Third, due to data limitations, subclinical heart failure and peripheral arterial disease were not assessed, which potentially resulted in an underestimation of advanced CKM stages. Finally, despite the overall large sample size, groups (eg, Black individuals, PPI user, participants with higher MgDS) still had limited numbers, sparse data bias is inevitable.

## Conclusion

In conclusion, our study revealed that a higher MgDS level (MgDS ≥ 2) was associated with increased odds of advanced CKM stages, with notable PIR differences. Our findings underscore the burden of advanced CKM stages among adults with magnesium deficiency and suggest that addressing magnesium-depleted nutritional status might be crucial for the prevention and treatment of advanced CKM stages.

## Supplementary Material

Supplementary tables.

## Figures and Tables

**Table 1:** Baseline characteristics of participants according to CKM syndrome stages.

Variables	Total (n=18038)	CKM Stages								
		Stage 0(n=1766)	Stage 1(n=3638)	Stage 2(n=9810)	Stage 3(n=1211)	Stage 4(n=1613)	*P* value	Nonadvanced (n=15214)	Advanced(n=2824)	*P* value
Age, y	45.86 ± 0.21	34.72 ± 0.35	39.56 ± 0.36	46.99 ± 0.22	70.91 ± 0.29	61.36 ± 0.39	< 0.0001	43.44 ± 0.21	64.78 ± 0.29	< 0.0001
Sex							< 0.0001			< 0.0001
Female	9115 (50.90)	1088 (63.70)	1992 (53.31)	4871 (48.66)	491 (44.60)	673 (42.08)		7951 (51.91)	1164 (42.98)	
Male	8923 (49.10)	678 (36.30)	1646 (46.69)	4939 (51.34)	720 (55.40)	940 (57.92)		7263 (48.09)	1660 (57.02)	
Race							< 0.0001			< 0.0001
Mexican American	3333 (8.22)	247 (5.98)	763 (10.73)	1922 (8.46)	205 (5.45)	196 (4.29)		2932 (8.67)	401 (4.70)	
Non-Hispanic Black	3645 (11.05)	267 (7.92)	710 (11.51)	1979 (11.07)	301 (14.60)	388 (12.83)		2956 (10.73)	689 (13.46)	
Non-Hispanic White	7838 (68.56)	930 (74.93)	1438 (64.32)	4119 (68.25)	542 (69.20)	809 (72.30)		6487 (68.23)	1351 (71.19)	
Other	3222 (12.17)	322 (11.17)	727 (13.43)	1790 (12.22)	163 (10.75)	220 (10.58)		2839 (12.37)	383 (10.64)	
Education							< 0.0001			< 0.0001
> High school	9301 (59.41)	1131 (69.22)	2109 (64.33)	4939 (57.97)	459 (45.88)	663 (46.50)		8179 (61.11)	1122 (46.28)	
High school	4120 (24.06)	354 (19.80)	748 (21.78)	2327 (25.17)	302 (28.04)	389 (27.77)		3429 (23.58)	691 (27.87)	
< High school	4607 (16.49)	281 (10.98)	780 (13.88)	2538 (16.86)	450 (26.08)	558 (25.72)		3599 (15.30)	1008 (25.85)	
PIR							< 0.0001			< 0.0001
<1.3	4904 (19.03)	417 (17.96)	933 (19.11)	2639 (20.00)	360 (24.55)	555 (27.94)		3989 (19.50)	915 (26.73)	
1.3-3.5	6331 (34.11)	587 (33.40)	1279 (36.50)	3386 (35.86)	503 (45.93)	576 (40.05)		5252 (35.67)	1079 (42.15)	
>3.5	5360 (40.58)	623 (48.64)	1162 (44.38)	2994 (44.14)	236 (29.52)	345 (32.01)		4779 (44.83)	581 (31.12)	
BMI, kg/m^2^	28.80 ± 0.09	21.88 ± 0.06	28.28 ± 0.11	30.26 ± 0.12	29.94 ± 0.24	30.78 ± 0.25	< 0.0001	28.59 ± 0.09	30.48 ± 0.19	< 0.0001
Waist circumference, cm	98.31 ± 0.22	79.23 ± 0.20	95.76 ± 0.25	102.17 ± 0.28	105.75 ± 0.54	106.48 ± 0.62	< 0.0001	97.34 ± 0.23	106.22 ± 0.45	< 0.0001
TGs, mmol/L	1.36 ± 0.01	0.80 ± 0.01	0.92 ± 0.01	1.60 ± 0.01	1.64 ± 0.03	1.60 ± 0.03	< 0.0001	1.32 ± 0.01	1.62 ± 0.02	< 0.0001
TC, mmol/L	5.02 ± 0.01	4.64 ± 0.03	4.87 ± 0.02	5.21 ± 0.02	4.87 ± 0.04	4.75 ± 0.04	< 0.0001	5.05 ± 0.01	4.79 ± 0.03	< 0.0001
HDL-c, mmol/L	1.39 ± 0.01	1.60 ± 0.01	1.46 ± 0.01	1.33 ± 0.01	1.30 ± 0.01	1.29 ± 0.02	< 0.0001	1.40 ± 0.01	1.30 ± 0.01	< 0.0001
LDL-c, mmol/L	3.00 ± 0.01	2.66 ± 0.03	2.98 ± 0.02	3.14 ± 0.01	2.82 ± 0.03	2.73 ± 0.03	< 0.0001	3.04 ± 0.01	2.76 ± 0.03	< 0.0001
eGFR, mL/min/1.73 m^2^	98.55 ± 0.28	107.57 ± 0.54	104.07 ± 0.45	98.64 ± 0.32	70.57 ± 0.79	81.41 ± 0.74	< 0.0001	101.23 ± 0.28	77.54 ± 0.59	< 0.0001
HOMA-IR	3.36 ± 0.04	1.31 ± 0.02	2.28 ± 0.04	3.87 ± 0.06	5.53 ± 0.28	5.09 ± 0.23	< 0.0001	3.12 ± 0.05	5.24 ± 0.16	< 0.0001
HbA_1c_, %	5.55 ± 0.01	5.12 ± 0.01	5.29 ± 0.01	5.62 ± 0.01	6.33 ± 0.05	6.08 ± 0.04	< 0.0001	5.47 ± 0.01	6.17 ± 0.03	< 0.0001
Ca, mg/dL	9.38 ± 0.01	9.41 ± 0.01	9.33 ± 0.01	9.38 ± 0.01	9.43 ± 0.02	9.38 ± 0.01	< 0.0001	9.37 ± 0.01	9.40 ± 0.01	0.03
Magnesium intake, mg/d	299.88 ± 1.95	311.26 ± 4.71	309.63 ± 3.91	299.34 ± 2.21	263.81 ± 4.64	275.43 ± 5.81	< 0.0001	303.54 ± 2.00	271.28 ± 4.23	< 0.0001
Current smoker							< 0.001			0.02
Yes	3876 (21.74)	405 (23.68)	725 (19.59)	2102 (21.66)	240 (19.86)	404 (26.49)		3232 (21.44)	644 (24.12)	
No	14162 (78.26)	1361 (76.32)	2913 (80.41)	7708 (78.34)	971 (80.14)	1209 (73.51)		11982 (78.56)	2180 (75.88)	
Current drinker							< 0.0001			< 0.0001
Yes	11900 (71.68)	1321 (82.50)	2620 (79.66)	6513 (75.64)	617 (57.47)	829 (60.95)		10454 (77.60)	1446 (59.69)	
No	5027 (23.13)	349 (17.50)	786 (20.34)	2718 (24.36)	519 (42.53)	655 (39.05)		3853 (22.40)	1174 (40.31)	
Physical activity							< 0.0001			0.24
Active	9154 (53.39)	999 (68.55)	2116 (72.94)	4904 (67.19)	473 (63.31)	662 (69.22)		8019 (68.84)	1135 (67.14)	
Inactive	4052 (24.35)	444 (31.45)	733 (27.06)	2291 (32.81)	268 (36.69)	316 (30.78)		3468 (31.16)	584 (32.86)	
Diuretics use							< 0.0001			< 0.0001
No	15685 (88.79)	1760 (99.62)	3618 (99.34)	8503 (86.98)	803 (64.99)	1001 (65.73)		13881 (91.78)	1804 (65.46)	
Yes	2351 (11.19)	6 (0.38)	20 (0.66)	1307 (13.02)	408 (35.01)	610 (34.27)		1333 (8.22)	1018 (34.54)	
PPI use							< 0.0001			< 0.0001
No	16542 (91.85)	1724 (97.71)	3504 (95.91)	9002 (90.98)	1017 (83.53)	1295 (80.84)		14230 (93.14)	2312 (81.81)	
Yes	1494 (8.15)	42 (2.29)	134 (4.09)	808 (9.02)	194 (16.47)	316 (19.16)		984 (6.86)	510 (18.19)	
Heavy drinking							< 0.0001			< 0.0001
No	15502 (83.15)	1457 (79.28)	3087 (82.40)	8340 (82.69)	1122 (90.90)	1496 (91.19)		12884 (82.14)	2618 (91.08)	
Yes	2536 (16.85)	309 (20.72)	551 (17.60)	1470 (17.31)	89 (9.10)	117 (8.81)		2330 (17.86)	206 (8.92)	
eGFR scores							< 0.0001			< 0.0001
0	11375 (63.11)	1461 (79.31)	2859 (73.81)	6395 (63.17)	196 (12.50)	464 (31.19)		10715 (68.05)	660 (24.51)	
1	5638 (32.50)	301 (20.56)	771 (25.98)	3157 (34.14)	623 (52.55)	786 (48.94)		4229 (30.23)	1409 (50.24)	
2	1023 (4.39)	4 (0.13)	8 (0.21)	258 (2.69)	392 (34.95)	361 (19.87)		270 (1.72)	753 (25.25)	
MgDS	0.77 ± 0.01	0.44 ± 0.02	0.49 ± 0.02	0.79 ± 0.01	1.83 ± 0.04	1.51 ± 0.04	< 0.0001	0.67 ± 0.01	1.62 ± 0.03	< 0.0001
MgDS categories							< 0.0001			< 0.0001
0	8545 (45.98)	1172 (61.29)	2351 (58.19)	4631 (44.19)	122 (6.98)	269 (18.43)		8154 (50.02)	391 (14.34)	
1	6044 (35.92)	525 (33.37)	1092 (35.20)	3493 (37.30)	402 (31.80)	532 (34.31)		5110 (36.23)	934 (33.42)	
2	2490 (13.53)	66 (5.17)	185 (6.27)	1337 (14.56)	425 (37.90)	477 (28.30)		1588 (11.21)	902 (31.72)	
≥3	959 (4.57)	3 (0.17)	10 (0.34)	349 (3.95)	262 (23.32)	335 (18.96)		362 (2.54)	597 (20.52)	

Abbreviations: PIR, poverty/income ratio; BMI, body mass index; TGs, triglycerides; TC, total cholesterol; HDL-c, high-density lipoprotein cholesterol; LDL-c, low-density lipoprotein cholesterol; eGFR, estimated glomerular filtration rate; HOMA-IR, homeostatic model assessment for insulin resistance; HbA_1c_, glycated hemoglobin A_1c_; Ca, serum calcium; PPI, proton pump inhibitor; MgDS, magnesium depletion score. Data are presented as mean ± SE or n (weighted %). Variable categories may not sum to 100% due to missing data.

**Table 2:** Multivariable logistic regression analysis of the association between MgDS categories and advanced CKM stages.

Variable	Model							
	Model 1			Model 2			Model 3	
	OR (95% CI)	*P*		OR (95% CI)	*P*		OR (95% CI)	*P*
MgDS categories								
0	Ref			Ref			Ref	
1	1.20(0.97,1.49)	0.09		1.26(1.00,1.57)	0.05		1.28(0.99,1.66)	0.06
2	2.31(1.82,2.94)	<0.0001		2.18(1.70,2.80)	<0.0001		2.33(1.74,3.14)	<0.0001
≥3	4.45(3.32,5.98)	<0.0001		3.68(2.68,5.06)	<0.0001		3.41(2.28,5.11)	<0.0001
*P* for trend		<0.0001			<0.0001			<0.0001

Model 1: adjusted for age, sex, race, education, PIR. Model 2: adjusted for age, sex, race, education, PIR, BMI, TG, HDL-c, LDL-c, HOMA-IR, HbA_1c_, Ca, magnesium intake. Model 3: adjusted for age, sex, race, education, PIR, BMI, TG, HDL-c, LDL-c, HOMA-IR, HbA_1c_, Ca, magnesium intake, current smoker, current drinker, physical activity. Abbreviations: OR, odds ratio; Ref, reference; PIR, poverty/income ratio; BMI, body mass index; TGs, triglycerides; HDL-c, high-density lipoprotein cholesterol; LDL-c, low-density lipoprotein cholesterol; HOMA-IR, homeostatic model assessment for insulin resistance; HbA_1c_, glycated hemoglobin A_1c_; Ca, serum calcium; PPI, proton pump inhibitor; MgDS, magnesium depletion score.
