# Supplementary material for: Association between Magnesium Depletion Score and Advanced Cardiovascular-Kidney-Metabolic Syndrome Stages in US Adults
Source: Int J Med Sci. 2025 Jun 23;22(12):3101–11. doi: 10.7150/ijms.115217 (PMC12244043; doi:10.7150/ijms.115217)
Supplement: Supplementary file 1 — Supplementary tables. [file ijmsv22p3101s1.pdf]

Association of Magnesium Depletion Score and Advanced Cardiovascular-Kidney-Metabolic Syndrome Stages in US Adults

Juan Tian[1*], Xiaoyu Ding[1*], Xiaoying Ren[1], Guang Wang[1], Wei Wang[2#], Jia Liu[1#]

[1]Department of Endocrinology, Beijing Chao-Yang Hospital, Capital Medical University, No. 8, Gongti South Road, Chaoyang District, Beijing 100020, China.

[2]Department of Urology, Beijing Chao-Yang Hospital, Capital Medical University, No. 8, Gongti South Road, Chaoyang District, Beijing 100020, China.

[#] **Corresponding authors**:

Wei Wang

Email: urodoctorwang@163.com

Department of Urology, Beijing Chao-Yang Hospital, Capital Medical University, No. 8, Gongti South Road, Chaoyang District, Beijing 100020, China.

Jia Liu

Email: liujiacy@ccmu.edu.cn

Department of Endocrinology, Beijing Chao-Yang Hospital, Capital Medical University, No. 8, Gongti South Road, Chaoyang District, Beijing 100020, China.

[*]These authors contributed equally to this work.

Table S1. Distribution of variables with missing data

Variables	Number of Missing	Percent (%)
Age	0	0
Sex	0	0
Race	0	0
Education	10	0.06
PIR	1443	8
BMI	136	0.75
Waist circumference	301	1.67
TGs	8	0.04
TC	0	0
HDL-c	0	0
LDL-c	0	0
eGFR	2	0.01
HOMA-IR	157	0.87
HbA1c	34	0.19
Ca	15	0.08
Magnesium intake	0	0
Current smoker	0	0
Current drinker	1111	6.16
Physical activity	4832	26.79
Diuretics use	2	0.01
PPI use	2	0.01
Heavy drinking	0	0
eGFR scores	2	0.01
MgDS	0	0
MgDS categories	0	0

Table S2. Collinearity Statistics

Variables	GVIF	Df	$GVIF^{1/2Df}$
MgDS	1.996418667	1	1.412946803
Age	1.539936383	1	1.240941732
Sex	1.884077140	1	1.372616895
Race	2.363815529	3	1.154167737
Education	2.573467048	2	1.266571329
PIR	2.254275986	2	1.225326345
BMI	1.370664611	1	1.170753864
TGs	1.929735592	1	1.389149233
HDL-c	1.914949827	1	1.383817122
LDL-c	1.184406028	1	1.088304198
HOMA-IR	1.658210989	1	1.287715415
HbA1c	1.435032869	1	1.197928574
Ca	1.469473711	1	1.212218508
Magnesium intake	1.559912368	1	1.248964518
Current smoker	1.571063080	1	1.253420552
Current drinker	1.566456323	1	1.251581529
Physical activity	1.203825614	1	1.097189871

Table S3. Baseline characteristics of participants after multiple imputation

Variables	Total (n=18038)	CKM Stages Stage 0 (n=1766)	Stage 1 (n=3638)	Stage 2 (n=9810)	Stage 3 (n=1211)	Stage 4 (n=1613)	*P* value	Nonadvanced (n=15214)	Advanced (n=2824)	*P* value
Age, y	45.86±0.21	34.72±0.35	39.56±0.36	46.99±0.22	70.91±0.29	61.36±0.39	< 0.0001	43.44±0.21	64.78±0.29	< 0.0001
Sex							< 0.0001			< 0.0001
Female	9115(50.90)	1088(63.70)	1992(53.31)	4871(48.66)	491(44.60)	673(42.08)		7951(51.91)	1164(42.98)	
Male	8923(49.10)	678(36.30)	1646(46.69)	4939(51.34)	720(55.40)	940(57.92)		7263(48.09)	1660(57.02)	
Race							< 0.0001			< 0.0001
Mexican American	3333(8.22)	247(5.98)	763(10.73)	1922(8.46)	205(5.45)	196(4.29)		2932(8.67)	401(4.70)	
Non-Hispanic Black	3645(11.05)	267(7.93)	710(11.51)	1979(11.07)	301(14.61)	388(12.83)		2956(10.74)	689(13.46)	
Non-Hispanic White	7838(68.56)	930(74.93)	1438(64.32)	4119(68.25)	542(69.20)	809(72.30)		6487(68.23)	1351(71.19)	
Other	3222(12.17)	322(11.17)	727(13.43)	1790(12.22)	163(10.75)	220(10.58)		2839(12.37)	383(10.64)	
Education							< 0.0001			< 0.0001
> High school	9307(59.43)	1131(69.22)	2110(64.35)	4942(57.97)	459(45.88)	665(46.51)		8183(61.12)	1124(46.29)	
High school	4121(24.06)	354(19.81)	748(21.77)	2328(25.16)	302(28.04)	389(27.76)		3430(23.58)	691(27.86)	
< High school	4610(16.50)	281(10.98)	780(13.88)	2540(16.87)	450(26.08)	559(25.73)		3601(15.31)	1009(25.85)	
PIR							< 0.0001			< 0.0001
<1.3	5418(20.71)	465(18.53)	1027(19.54)	2905(20.34)	408(24.99)	613(28.24)		4397(19.89)	1021(27.08)	
1.3–3.5	6886(36.54)	631(33.45)	1375(36.41)	3710(36.25)	544(45.33)	626(39.55)		5716(35.90)	1170(41.62)	
>3.5	5734(42.75)	670(48.02)	1236(44.06)	3195(43.40)	259(29.68)	374(32.21)		5101(44.21)	633(31.31)	
BMI, kg/m^2	28.82±0.09	21.88±0.06	28.28±0.11	30.28±0.12	29.92±0.24	30.80±0.25	< 0.0001	28.61±0.09	30.49±0.19	< 0.0001
Waist circumference, cm	98.47±0.22	79.23±0.20	95.76±0.25	102.27±0.28	106.03±0.56	106.90±0.60	< 0.0001	97.43±0.23	106.59±0.45	< 0.0001
TGs, mmol/L	1.36±0.01	0.80±0.01	0.92±0.01	1.60±0.01	1.64±0.03	1.60±0.03	< 0.0001	1.32±0.01	1.62±0.02	< 0.0001
TC, mmol/L	5.02±0.01	4.64±0.03	4.87±0.02	5.21±0.02	4.87±0.04	4.75±0.04	< 0.0001	5.05±0.01	4.79±0.03	< 0.0001
HDL-c, mmol/L	1.39±0.01	1.60±0.01	1.46±0.01	1.33±0.01	1.30±0.01	1.29±0.02	< 0.0001	1.40±0.01	1.30±0.01	< 0.0001
LDL-c, mmol/L	3.00±0.01	2.66±0.03	2.98±0.02	3.14±0.01	2.82±0.03	2.73±0.03	< 0.0001	3.04±0.01	2.76±0.03	< 0.0001

Variables	Total (n=18038)	CKM Stages Stage 0 (n=1766)	Stage 1 (n=3638)	Stage 2 (n=9810)	Stage 3 (n=1211)	Stage 4 (n=1613)	***P* value**	Nonadvanced (n=15214)	Advanced (n=2824)	***P* value**
eGFR, mL/min/1.73 m^2	98.55±0.28	107.57±0.54	104.07±0.45	98.64±0.32	70.57±0.79	81.42±0.74	< 0.0001	101.23±0.28	77.54±0.59	< 0.0001
HOMA-IR	3.36±0.04	1.33±0.02	2.30±0.04	3.85±0.06	5.53±0.28	5.08±0.23	< 0.0001	3.12±0.05	5.24±0.16	< 0.0001
HbA1c, %	5.55±0.01	5.12±0.01	5.29±0.01	5.62±0.01	6.33±0.05	6.08±0.04	< 0.0001	5.47±0.01	6.17±0.03	< 0.0001
Ca, mg/dL	9.38±0.01	9.41±0.01	9.33±0.01	9.38±0.01	9.43±0.02	9.38±0.01	< 0.0001	9.37±0.01	9.40±0.01	0.03
Magnesium intake, mg/d	299.88±1.95	311.26±4.71	309.63±3.91	299.34±2.21	263.81±4.64	275.43±5.81	< 0.0001	303.54±2.00	271.28±4.23	< 0.0001
Current smoker							< 0.001			0.02
Yes	3876(21.74)	405(23.68)	725(19.59)	2102(21.66)	240(19.86)	404(26.49)		3232(21.44)	644(24.12)	
No	14162(78.26)	1361(76.32)	2913(80.41)	7708(78.34)	971(80.14)	1209(73.51)		11982(78.56)	2180(75.88)	
Current drinker							< 0.0001			< 0.0001
Yes	12635(75.35)	1393(82.24)	2795(79.43)	6893(75.39)	656(57.53)	898(60.99)		11081(77.34)	1554(59.76)	
No	5403(24.65)	373(17.76)	843(20.57)	2917(24.61)	555(42.47)	715(39.01)		4133(22.66)	1270(40.24)	
Physical activity							< 0.0001			0.08
Active	12213(67.29)	1192(67.04)	2649(71.53)	6544(66.05)	755(61.80)	1073(67.41)		10385(67.53)	1828(65.40)	
Inactive	5825(32.71)	574(32.96)	989(28.47)	3266(33.95)	456(38.20)	540(32.59)		4829(32.47)	996(34.60)	
Diuretics use							< 0.0001			< 0.0001
No	15685(88.79)	1760(99.62)	3618(99.34)	8503(86.98)	803(64.99)	1001(65.61)		13881(91.78)	1804(65.39)	
Yes	2353(11.21)	6(0.38)	20(0.66)	1307(13.02)	408(35.01)	612(34.39)		1333(8.22)	1020(34.61)	
PPI use							< 0.0001			< 0.0001
No	16544(91.85)	1724(97.71)	3504(95.91)	9002(90.98)	1017(83.53)	1297(80.88)		14230(93.14)	2314(81.83)	
Yes	1494(8.15)	42(2.29)	134(4.09)	808(9.02)	194(16.47)	316(19.12)		984(6.86)	510(18.17)	
Heavy drinking							< 0.0001			< 0.0001
No	15502(83.15)	1457(79.28)	3087(82.40)	8340(82.69)	1122(90.90)	1496(91.19)		12884(82.14)	2618(91.08)	
Yes	2536(16.85)	309(20.72)	551(17.60)	1470(17.31)	89(9.10)	117(8.81)		2330(17.86)	206(8.92)	
eGFR scores							< 0.0001			< 0.0001
0	11375(63.11)	1461(79.31)	2859(73.81)	6395(63.17)	196(12.50)	464(31.18)		10715(68.05)	660(24.51)	

Variables	Total	CKM Stages								
	(n=18038)	Stage 0 (n=1766)	Stage 1 (n=3638)	Stage 2 (n=9810)	Stage 3 (n=1211)	Stage 4 (n=1613)	*P* value	Nonadvanced (n=15214)	Advanced (n=2824)	*P* value
1	5640(32.50)	301(20.56)	771(25.98)	3157(34.14)	623(52.55)	788(48.96)		4229(30.23)	1411(50.25)	
2	1023(4.39)	4(0.13)	8(0.21)	258(2.69)	392(34.95)	361(19.86)		270(1.72)	753(25.25)	
MgDS	0.77±0.01	0.44±0.02	0.49±0.02	0.79±0.01	1.83±0.04	1.51±0.04	< 0.0001	0.67±0.01	1.62±0.03	< 0.0001
MgDS categories							< 0.0001			< 0.0001
0	8545(45.98)	1172(61.29)	2351(58.19)	4631(44.19)	122(6.98)	269(18.43)		8154(50.02)	391(14.34)	
1	6044(35.91)	525(33.37)	1092(35.20)	3493(37.30)	402(31.81)	532(34.31)		5110(36.23)	934(33.42)	
2	2490(13.53)	66(5.17)	185(6.27)	1337(14.56)	425(37.90)	477(28.30)		1588(11.21)	902(31.73)	
≥3	959(4.57)	3(0.17)	10(0.34)	349(3.96)	262(23.32)	335(18.96)		362(2.54)	597(20.52)	

Abbreviations: PIR, poverty/income ratio; BMI, body mass index; TGs, triglycerides; TC, total cholesterol; HDL-c, high-density lipoprotein cholesterol; LDL-c, low-density lipoprotein cholesterol; eGFR, estimated glomerular filtration rate; HOMA-IR, homeostatic model assessment for insulin resistance; HbA1c, glycated hemoglobin A1c; Ca, serum calcium; PPI, proton pump inhibitor; MgDS, magnesium depletion score. Data are presented as mean ± SE or n (weighted %).

Table S4. Association between MgDS and advanced CKM stages after multiple imputation

Variable	**Model**					
	Model 1		Model 2		Model 3	
	OR (95% CI)	*P*	OR (95% CI)	*P*	OR (95% CI)	*P*
MgDS categories						
0	Ref		Ref		Ref	
1	1.25(1.02,1.53)	0.03	1.34(1.08,1.66)	0.01	1.32(1.07,1.64)	0.01
2	2.34(1.86,2.94)	<0.0001	2.32(1.83,2.93)	<0.0001	2.36(1.87,2.97)	<0.0001
≥3	4.59(3.45,6.11)	<0.0001	3.99(2.95,5.39)	<0.0001	4.16(3.06,5.64)	<0.0001
P for trend		<0.0001		<0.0001		<0.0001

Model 1: adjusted for age, sex, race, education, PIR.

Model 2: adjusted for age, sex, race, education, PIR, BMI, TG, HDL-c, LDL-c, HOMA-IR, HbA1c, Ca, magnesium intake.

Model 3: adjusted for age, sex, race, education, PIR, BMI, TG, HDL-c, LDL-c, HOMA-IR, HbA1c, Ca, magnesium intake, current smoker, current drinker, physical activity.

Abbreviations: OR, odds ratio; Ref, reference; PIR, poverty/income ratio; BMI, body mass index; TGs, triglycerides; HDL-c, high-density lipoprotein cholesterol; LDL-c, low-density lipoprotein cholesterol; HOMA-IR, homeostatic model assessment for insulin resistance; HbA1c, glycated hemoglobin A1c; Ca, serum calcium; MgDS, magnesium depletion score.

Table S5. Baseline characteristics of participants after excluding those with any missing values

Variables	Total (n=11277)	CKM Stages Stage 0 (n=1250)	Stage 1 (n=2441)	Stage 2 (n=6157)	Stage 3 (n=613)	Stage 4 (n=816)	*P* value	Nonadvanced (n=9848)	Advanced (n=1429)	*P* value
Age, y	44.76±0.25	34.94±0.39	39.49±0.43	46.18±0.29	70.90±0.41	60.96±0.53	< 0.0001	42.79±0.27	64.49±0.40	< 0.0001
Sex							< 0.0001			< 0.0001
Female	5296(48.03)	759(63.19)	1263(50.62)	2784(44.73)	196(38.26)	294(38.24)		4806(49.01)	490(38.25)	
Male	5981(51.97)	491(36.81)	1178(49.38)	3373(55.27)	417(61.74)	522(61.76)		5042(50.99)	939(61.75)	
Race							< 0.0001			< 0.0001
Mexican American	1817(6.92)	135(4.42)	438(8.93)	1057(7.19)	95(4.47)	92(3.66)		1630(7.22)	187(3.95)	
Non-Hispanic Black	2081(9.49)	163(6.53)	450(10.09)	1151(9.67)	139(11.98)	178(11.03)		1764(9.30)	317(11.37)	
Non-Hispanic White	5486(72.74)	743(80.01)	1105(69.26)	2887(71.96)	302(73.24)	449(76.27)		4735(72.49)	751(75.19)	
Other	1893(10.85)	209(9.04)	448(11.72)	1062(11.18)	77(10.31)	97(9.03)		1719(10.99)	174(9.49)	
Education							< 0.0001			< 0.0001
> High school	6431(63.77)	860(72.79)	1517(67.31)	3398(61.94)	269(50.39)	387(52.96)		5775(64.94)	656(52.05)	
High school	2585(23.28)	240(18.61)	501(21.37)	1478(24.60)	164(29.80)	202(25.94)		2219(22.88)	366(27.31)	
< High school	2261(12.95)	150(8.60)	423(11.33)	1281(13.46)	180(19.81)	227(21.10)		1854(12.18)	407(20.64)	
PIR							< 0.0001			< 0.0001
<1.3	2961(17.48)	289(15.64)	602(16.82)	1638(17.59)	169(19.66)	263(22.17)		2529(17.10)	432(21.28)	
1.3–3.5	4203(35.24)	440(32.65)	917(35.84)	2254(34.61)	290(47.47)	302(37.97)		3611(34.63)	592(41.34)	
>3.5	4113(47.28)	521(51.70)	922(47.34)	2265(47.80)	154(32.86)	251(39.86)		3708(48.27)	405(37.37)	
BMI, kg/m^2	28.43±0.10	21.89±0.07	28.20±0.13	29.93±0.13	29.26±0.29	30.40±0.30	< 0.0001	28.28±0.10	30.00±0.21	< 0.0001
Waist circumference, cm	97.61±0.25	79.28±0.23	95.60±0.32	101.76±0.31	104.95±0.71	106.10±0.72	< 0.0001	96.80±0.27	105.69±0.51	< 0.0001
TGs, mmol/L	1.33±0.01	0.79±0.01	0.92±0.01	1.60±0.02	1.59±0.04	1.56±0.04	< 0.0001	1.31±0.01	1.57±0.03	< 0.0001
TC, mmol/L	5.03±0.01	4.65±0.03	4.87±0.02	5.23±0.02	4.93±0.05	4.73±0.05	< 0.0001	5.05±0.01	4.80±0.04	< 0.0001
HDL-c, mmol/L	1.40±0.01	1.61±0.01	1.46±0.01	1.33±0.01	1.30±0.02	1.30±0.02	< 0.0001	1.41±0.01	1.30±0.02	< 0.0001
LDL-c, mmol/L	3.02±0.01	2.66±0.03	2.98±0.02	3.17±0.02	2.90±0.05	2.71±0.04	< 0.0001	3.04±0.01	2.78±0.04	< 0.0001

Variables	Total (n=11277)	CKM Stages Stage 0 (n=1250)	Stage 1 (n=2441)	Stage 2 (n=6157)	Stage 3 (n=613)	Stage 4 (n=816)	*P* value	Nonadvanced (n=9848)	Advanced (n=1429)	*P* value
eGFR, mL/min/1.73 m^2	99.05±0.32	106.28±0.62	103.49±0.49	98.75±0.39	71.49±1.02	82.52±0.83	< 0.0001	101.09±0.33	78.60±0.63	< 0.0001
HOMA-IR	3.06±0.05	1.26±0.02	2.21±0.04	3.65±0.07	4.11±0.20	4.67±0.22	< 0.0001	2.92±0.05	4.47±0.14	< 0.0001
HbA1c, %	5.49±0.01	5.12±0.01	5.28±0.01	5.59±0.01	6.21±0.06	5.96±0.05	< 0.0001	5.44±0.01	6.05±0.04	< 0.0001
Ca, mg/dL	9.38±0.01	9.40±0.01	9.34±0.01	9.39±0.01	9.44±0.02	9.40±0.02	< 0.0001	9.38±0.01	9.41±0.02	0.02
Magnesium intake, mg/d	310.19±2.29	320.14±5.46	319.33±4.89	308.00±2.84	273.80±6.72	291.26±6.42	< 0.0001	312.71±2.38	285.06±4.68	< 0.0001
Current smoker							0.01			0.36
Yes	2394(21.23)	275(22.93)	481(19.18)	1333(21.46)	112(17.03)	193(25.54)		2089(21.11)	305(22.52)	
No	8883(78.77)	975(77.07)	1960(80.82)	4824(78.54)	501(82.97)	623(74.46)		7759(78.89)	1124(77.48)	
Current drinker							< 0.0001			< 0.0001
Yes	8371(78.61)	1021(84.46)	1921(80.90)	4549(78.41)	375(63.56)	505(66.00)		7491(79.95)	880(65.13)	
No	2906(21.39)	229(15.54)	520(19.10)	1608(21.59)	238(36.44)	311(34.00)		2357(20.05)	549(34.87)	
Physical activity							< 0.0001			0.06
Active	7756(68.36)	868(68.71)	1800(72.76)	4165(66.84)	380(62.46)	543(67.37)		6833(68.63)	923(65.63)	
Inactive	3521(31.64)	382(31.29)	641(27.24)	1992(33.16)	233(37.54)	273(32.63)		3015(31.37)	506(34.37)	
Diuretics use							< 0.0001			< 0.0001
No	10024(90.23)	1247(99.78)	2425(99.18)	5407(87.92)	413(65.06)	532(67.81)		9079(92.57)	945(66.83)	
Yes	1253(9.77)	3(0.22)	16(0.82)	750(12.08)	200(34.94)	284(32.19)		769(7.43)	484(33.17)	
PPI use							< 0.0001			< 0.0001
No	10462(92.72)	1223(97.97)	2355(96.14)	5693(91.46)	523(85.08)	668(82.84)		9271(93.63)	1191(83.64)	
Yes	815(7.28)	27(2.03)	86(3.86)	464(8.54)	90(14.92)	148(17.16)		577(6.37)	238(16.36)	
Heavy drinking							< 0.0001			< 0.0001
No	9488(81.28)	1003(76.69)	2031(80.53)	5141(81.14)	563(90.58)	750(91.16)		8175(80.31)	1313(90.95)	
Yes	1789(18.72)	247(23.31)	410(19.47)	1016(18.86)	50(9.42)	66(8.84)		1673(19.69)	116(9.05)	
eGFR scores							< 0.0001			< 0.0001
0	7213(63.52)	1013(77.79)	1891(72.69)	3977(62.78)	99(11.85)	233(29.35)		6881(67.56)	332(23.13)	

Variables	Total (n=11277)	CKM Stages Stage 0 (n=1250)	Stage 1 (n=2441)	Stage 2 (n=6157)	Stage 3 (n=613)	Stage 4 (n=816)	*P* value	Nonadvanced (n=9848)	Advanced (n=1429)	*P* value
1	3573(33.00)	236(22.14)	545(27.09)	2030(34.79)	333(54.81)	429(53.16)		2811(30.92)	762(53.74)	
2	491(3.48)	1(0.07)	5(0.22)	150(2.44)	181(33.34)	154(17.50)		156(1.52)	335(23.12)	
MgDS	0.76±0.01	0.48±0.02	0.52±0.02	0.79±0.02	1.81±0.05	1.46±0.04	< 0.0001	0.67±0.01	1.59±0.03	< 0.0001
MgDS categories							< 0.0001			< 0.0001
0	5376(45.64)	788(57.83)	1520(55.71)	2864(43.58)	67(6.93)	137(17.79)		5172(48.81)	204(13.93)	
1	3954(37.59)	411(36.65)	782(37.24)	2263(38.36)	205(32.23)	293(37.04)		3456(37.82)	498(35.34)	
2	1459(12.77)	49(5.37)	132(6.70)	820(14.02)	219(38.92)	239(28.11)		1001(10.85)	458(31.95)	
≥3	488(3.99)	2(0.15)	7(0.35)	210(4.04)	122(21.92)	147(17.05)		219(2.52)	269(18.78)	

Abbreviations: PIR, poverty/income ratio; BMI, body mass index; TGs, triglycerides; TC, total cholesterol; HDL-c, high-density lipoprotein cholesterol; LDL-c, low-density lipoprotein cholesterol; eGFR, estimated glomerular filtration rate; HOMA-IR, homeostatic model assessment for insulin resistance; HbA1c, glycated hemoglobin A1c; Ca, serum calcium; PPI, proton pump inhibitor; MgDS, magnesium depletion score. Data are presented as mean ± SE or n (weighted %).

Table S6. Association between MgDS and advanced CKM stages after excluding individuals with any missing values

Variable	**Model**					
	Model 1		Model 2		Model 3	
	OR (95% CI)	*P*	OR (95% CI)	*P*	OR (95% CI)	*P*
MgDS categories						
0	Ref		Ref		Ref	
1	1.23(0.96,1.57)	0.1	1.31(1.01,1.70)	0.04	1.30(1.01,1.69)	0.05
2	2.29(1.73,3.04)	<0.0001	2.32(1.72,3.14)	<0.0001	2.37(1.76,3.18)	<0.0001
≥3	3.85(2.69,5.52)	<0.0001	3.37(2.26,5.02)	<0.0001	3.48(2.33,5.21)	<0.0001
P for trend		<0.0001		<0.0001		<0.0001

Model 1: adjusted for age, sex, race, education, PIR.

Model 2: adjusted for age, sex, race, education, PIR, BMI, TG, HDL-c, LDL-c, HOMA-IR, HbA1c, Ca, magnesium intake.

Model 3: adjusted for age, sex, race, education, PIR, BMI, TG, HDL-c, LDL-c, HOMA-IR, HbA1c, Ca, magnesium intake, current smoker, current drinker, physical activity.

Abbreviations: OR, odds ratio; Ref, reference; PIR, poverty/income ratio; BMI, body mass index; TGs, triglycerides; HDL-c, high-density lipoprotein cholesterol; LDL-c, low-density lipoprotein cholesterol; HOMA-IR, homeostatic model assessment for insulin resistance; HbA1c, glycated hemoglobin A1c; Ca, serum calcium; MgDS, magnesium depletion score.

Table S7 Knots selection of restricted cubic spline analysis

Number of knots	Akaike information criterion	Bayesian information criterion	Final selected number of knots
3	4494.757	4665.542	3
4	4493.622	4671.833	
5	4493.622*	4671.833*	
6	4493.622*	4671.833*	

*rcspline.eval() limits the number of interior knots to at most (number of unique predictor values – 2). Since MgDS has only 6 unique values, the function caps interior knots at 4. Thus, specifying the number of knots ≥ 4 yields the same 4 knots and identical model results.
